# Influence of Antibiotic-Loaded Acrylic Bone Cement Composition on Drug Release Behavior and Mechanism

**DOI:** 10.3390/polym13142240

**Published:** 2021-07-08

**Authors:** I-Cheng Chen, Chen-Ying Su, Wei-Han Nien, Tzu-Tien Huang, Chang-Hung Huang, Yung-Chang Lu, Yu-Jen Chen, Gwo-Che Huang, Hsu-Wei Fang

**Affiliations:** 1 Accelerator for Happiness and Health Industry, National Taipei University of Technology, No. 1, Sec. 3, Zhongxiao E. Rd., Taipei 10608, Taiwan; icchen.ntut@mail.ntut.edu.tw; 2 Department of Chemical Engineering and Biotechnology, National Taipei University of Technology, No. 1, Sec. 3, Zhongxiao E. Rd., Taipei 10608, Taiwan; chenying.su@mail.ntut.edu.tw (C.-Y.S.); enjoylive00@yahoo.com.tw (W.-H.N.); a350163@gmail.com (T.-T.H.); 3 Department of Medical Research, Biomechanics Research Laboratory, Mackay Memorial Hospital, New Taipei City 251020, Taiwan; changhung0812@gmail.com (C.-H.H.); yungchanglu@homail.com (Y.-C.L.); 4 Institute of Biomedical Engineering, National Yang-Ming University, Taipei 11221, Taiwan; 5 Department of Orthopaedic Surgery, Mackay Memorial Hospital, Taipei 10491, Taiwan; 6 Department of Radiation Oncology, MacKay Memorial Hospital, Taipei 10491, Taiwan; chenmdphd@gmail.com; 7 Institute of Biomedical Engineering and Nanomedicine, National Health Research Institutes, No. 35, Keyan Road, Zhunan Town, Miaoli County 35053, Taiwan

**Keywords:** infection, antibiotic-loaded bone cement, antibacterial, drug delivery, drug release, biomaterials

## Abstract

Periprosthetic joint infection (PJI) is a devastating complication after total joint replacement with considerable morbidity and large economic burdens. Antibiotic-Loaded Bone Cement (ALBC) has been developed as a valuable tool for local administration and is becoming one of the most effective methods for the prevention and treatment of orthopedic infections. Controlling antibiotic release from ALBC is critical to achieve effective infection control, however, the antibiotic elution rates are generally low, and the mechanisms are poorly understood. Thus, the present study aims to investigate the effects of the basic acrylic bone cement components, including liquid/powder (monomer-to-polymer) ratios, radiopacifier, initiator, and doses of antibiotics on the porosity, antibiotic elution rates and mechanical properties of polymethylmethacrylate (PMMA) based ALBC. The obtained results from the in vitro studies suggested that a reduction in the liquid/powder ratio and an increase in the radiopacifier ratio and gentamicin doses led to increased porosity and release of antibiotic, while the initiator ratio exerted no effect on elution rates. In conclusion, we hope that by varying the composition of ALBC, we could considerably enhance the antibiotic elution rates by increasing porosity, while maintaining an adequate mechanical strength of the bone cements. This finding might provide insights into controlling antibiotic release from ALBC to achieve effective infection control after total joint replacement surgery.

## 1. Introduction

Demand for arthroplasties has increased in recent years and is estimated to grow drastically by 174% and 673% for primary total hip arthroplasties (THA) and primary total knee arthroplasties (TKA), respectively, by 2030 [1]. Despite great survival rates and improved outcomes of arthroplasties, various failures and complications were reported, including periprosthetic joint infection (PJI), which is one of the most common failure mechanisms. PJI occurs following primary THA and TKA, with rates ranging between 0.3% and 1.9%, and up to 10% in revision cases [2,3]. PJI is a devastating failure with considerable morbidity and large economic burdens, both for affected patients and the healthcare system. To reduce infection, antibiotic-loaded bone cement (ALBC) has been developed as a valuable tool and is becoming one of the most effective methods for the prevention and treatment of orthopedic infections [4].

Bone cement is a vital component of total joint arthroplasty (TJA) to fix prostheses in joint replacements and delivers antibiotics locally. Local delivery of antibiotics avoids toxicity caused by systemic administration and ALBC provides high concentration of antibiotics over a period of time at the implant site to be more effective against local infections [5,6]. The efficiency of the release of antibiotics from bone cement is an important factor determining the antibacterial activity of ALBC. As controlling antibiotic release from ALBC is critical to the clinical efficacy and the release mechanisms are poorly understood, several studies have been conducted with different types and doses of antibiotics, mixing methods, temperatures at mixing, a combination of different antibiotics, and other additives, such as xylitol, in order to enhance antibiotic elution capability [7,8,9,10]. With recent advancements in nanotechnology, drugs could also be controlled and delivered using biomimetic nanoparticles or biodegradable polymers [11,12,13,14]. Despite the fact that some positive clinical outcomes of ALBC were revealed, the elution rate of the loaded antibiotics from bone cement were generally low and varied, with different studies, ranging from 4% to 17% [4,6,10,15].

Bone cements are applied widely in surgery as bone implants and are commonly offered as two components: a powder and a liquid. The powder component consists of polymethylmethacrylate (PMMA) copolymer, initiator (benzoyl peroxide, BPO), radiopacifier (e.g., Barium sulphate, BaSO_4_) and additives, while the liquid component contains methylmethacrylate (MMA) monomer, activator (*N*,*N*-dimethyl-*p*-toluidine, DMPT), inhibitors and additives. When the two components are mixed, initiators and activators form radicals and initiate polymerization to generate polymer chains [16]. PMMA bone cement is mainly used for the fixation of endoprostheses. The sufficient strength to endure considerable stresses is one of the most important concerns for achieving a stable fixation and long-term stability of the implant. It was hypothesized that altering the ratio of cement components would affect the material properties of the bone cement, which, in turn, might affect the elution rates of the loaded antibiotics from the bone cement. Thus, the aim of this study is to explore the material behavior of the gentamicin-loaded acrylic bone cement cured with different bone cement components and maximize the anti-bacterial effects of ALBC by increasing the antibiotic elution rates. The effects of different bone cement components on antibiotic elution were investigated by systematic comparison of different liquid/powder ratios, radiopacifier ratios, initiator ratios and antibiotic doses. The morphology, porosity, and mechanical properties (i.e., compressive strength) of these tested cements were also determined and discussed. The obtained outcome from the present study may provide valuable information for the development of ALBC.

## 2. Materials and Methods

### 2.1. Material Composition and Mixing Procedure

The components of basic PMMA bone cement were listed in Table 1 (PMMA, MMA, BPO and DMPT: Chain Bridge Industry Co. Ltd., New Taipei City, Taiwan; BaSO_4_: Echo Chemical Co. Ltd., Miaoli, Taiwan; Gentamicin: Sigma, St. Louis, MO, USA). Table 2 showed the composition from tested bone cements with different parameters (liquid/powder ratios, radiopacifier, initiator and antibiotic) in detail. In general, to prepare the bone cements, 4 g of polymer powder was mixed with 2 mL of liquid monomer and 0.1 g of powdered gentamicin for 2 min by hand at atmospheric conditions. Upon reaching the dough phase, the cement was spatulated into stainless steel molds and allowed to cure. The resulting cylindrical samples had a diameter of 5 mm and a height of 3 mm for the bacterial inhibition test and a diameter of 6 mm and a height of 12 mm for other tests performed in this study.

### 2.2. Scanning Electronic Microscopy Imaging

Representative images and particle sizes from different cement specimens (*n* = 3) were obtained by scanning electronic microscopy (SEM) using S-3000H microscope (Htachi, Tokyo, Japan) under low vacuum conditions. Each specimen was covered with gold by a sputter coater (Ion Sputter E101, Hitachi, Tokyo, Japan).

### 2.3. Cell Viability

We purchased L929 cells (mouse fibroblasts, Strains number BCRC 60091) from Food Industry Research and Development Institute, Hsinchu, Taiwan. The cells were routinely maintained in Modified Eagle Medium (MEM, Gibco, Thermo Fisher Scientific Inc., Waltham, MA, USA) and supplemented with 10% fetal bovine serum (FBS, Invitrogen, Waltham, MA, USA) at 37 °C under 5% CO_2_ and 95% relative humidity.

In vitro cell viability testing of bone cement samples was examined according to ISO10993-5. Briefly, 2 g of bone cement was extracted in 10 mL of MEM at 37 °C for 24 h (*n* = 3). L929 cells were plated in a density of 1 × 10^4^ cells/well onto a 96-well culture plate in culture medium at 37 °C overnight. After removing culture medium on the next day, cells were washed with phosphate-buffer saline (PBS) and cultured in MEM supplemented with 1% FBS containing bone cement extract (0.2 g/mL). 3-(4,5-dimethylthiazol-2-yl)-2,5-diphenyltetrazolium bromide (MTT, Sigma, St. Louis, MO, USA) solution was then added into the medium, and cells were incubated at 37 °C for 2 h. The samples were read by an Enzyme-linked immunosorbent assay (ELISA) reader (Tecan, Männedorf, Switzerland) with a wavelength of 570 nm to obtain OD values. Cell viability higher than 70% was considered good biocompatibility.

### 2.4. Mechanical Compression Testing

The compressive strength of cement was examined in accordance with ASTM F451 standards with a minimum of 70 MPa as a threshold. Cylindrical samples (6 mm × 12 mm) were prepared and soaked in PBS at 37 °C for 28 days prior to compressive testing and the determination of compressive properties was performed at a loading rate of 20 mm/min with a universal mechanical testing machine (Chun Yen Testing Machines Co., Ltd., Taichung, Taiwan). Cement specimens were tested in triplicate.

### 2.5. Bioassay of Antibiotic Activity

**Figure 1:**
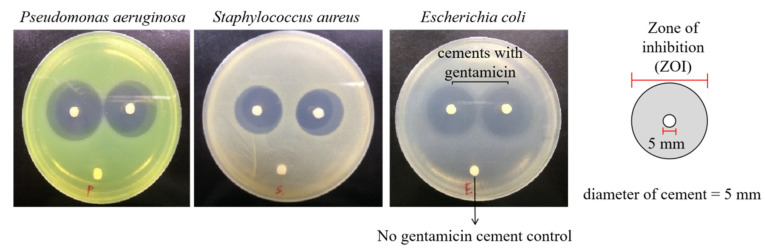
Representative photos of agar disk diffusion bioassay. ZOI of *Pseudomonas aeruginosa*, *Staphylococcus aureus* and *Escherichia coli* was measured as indicated in the illustration. The diameter of the bone cement disc was 5 mm.

The bioactivity of gentamicin eluted from bone cement was estimated using an agar disk diffusion bioassay. Briefly, basic PMMA bone cement discs containing gentamicin were prepared as described in Section 2.1. (0.1 g gentamicin in 4 g PMMA powder component/2 mL liquid component, Table 1) with a diameter of 5 mm and height of 3 mm. The tested cement discs were placed on Mueller Hinton agar plates (Sigma, St. Louis, MO, USA) inoculated with *Pseudomonas aeruginosa* (ATCC27853), *Staphylococcus aureus* (ATCC25923) or *Escherichia coli* (ATCC25922) in a density of 1 × 10^6^/mL and incubated at 30 °C (*n* = 4). The tested cements were transferred to new agar plates inoculated with bacteria every 24 h and the diameter of the inhibition zones (zone of inhibition, ZOI; Figure 1) on day 1, 2, 3, 7 and 14 was measured. Bone cement without antibiotic was used as a negative control (ZOI = 5 mm).

### 2.6. Porosity and Pore Size Distribution

Cylindrical samples (6 mm × 12 mm) were submerged in mercury and external pressure 12,500 Psi was required to push the mercury into a cylindrical pore. Pore sizes was obtained, and the porosity percentage of the cements was determined using Aquapore porosimeter (AAQ-30K-A-1, Porous Materials Inc, Ithaca, NY, USA). Samples were tested in triplicate.

### 2.7. Antibiotic Elution

Tested cylindrical samples (6 mm × 12 mm) were prepared as mentioned in Section 2.1. (Table 2) and soaked in 10 mL of PBS at 37 °C for 0.25, 1, 2, 3, 4, 7, 11, 15 and 28 days. To determine the antibiotic release from the bone cements, the eluent was collected at each time point and samples were placed into fresh PBS. The concentration of gentamicin present in the collected eluent was determined by reacting with o-phthaldialdehyde (OPA) reagent (Sigma, St. Louis, MO, USA) following the study by Cabanillas et al. [17]. Briefly, the gentamicin eluent, OPA reagent, and isopropanol were mixed in equal proportions and stored for 30 min at room temperature. When OPA reacted with amino groups of gentamicin, the resultant chromophoric products were measured at 332 nm using an ELISA reader (Tecan, Männedorf, Switzerland). Gentamicin concentrations in the samples were calculated with a calibration curve. The percentages of gentamicin released were calculated with respect to the total amount incorporated and compared with the gentamicin release from tested bone cements. Samples were tested in triplicate.

## 3. Results

### 3.1. PMMA Bone Cement Composite and Antibacterial Properties

**Figure 2:**
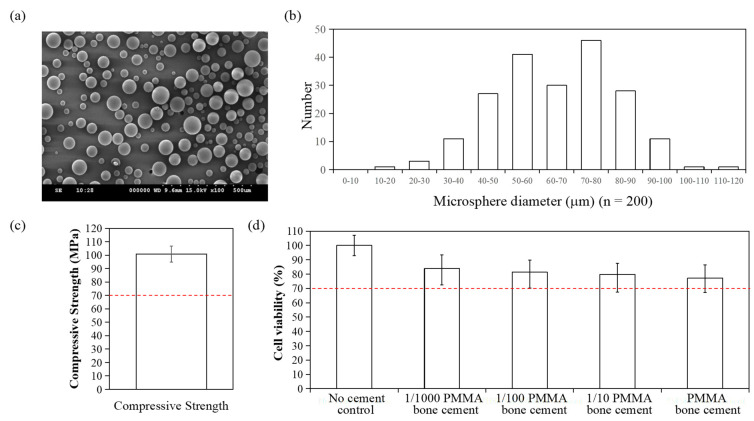
Characterization of PMMA bone cement. (**a**) SEM image of PMMA bone cement. (**b**) Particle diameter distribution of PMMA. (**c**) Compressive strength of PMMA bone cement. (**d**) Cell viability of L929 cells measured by MTT assay for evaluating the biocompatibility of PMMA bone cement. The dashed lines indicate the ISO standard for compressive strength (70 MPa) and cell viability (70%).

In this study, a basic formulation of PMMA bone cement was first determined. To obtain basic PMMA bone cement, suitable powder component (containing PMMA polymer, initiator and radiopacifier) and liquid component (MMA monomer and activator) were selected, as shown in Table 1. The molecular weight of PMMA used in this study is 519,994 g/mol and the average particle size is 64.88 µm, ranging from 19.8~113.25 µm (Figure 2a,b). The bone cement preparation began by mixing two sterile components and the produced bone cement presented good handling characteristics and mechanical properties that the compression strength was 100.89 MPa, superior to the 70 MPa of ISO 5883 standards (Figure 2c). The extract of basic bone cement showed low cytotoxicity and demonstrated good biocompatibility, as shown in Figure 2d.

**Figure 3:**
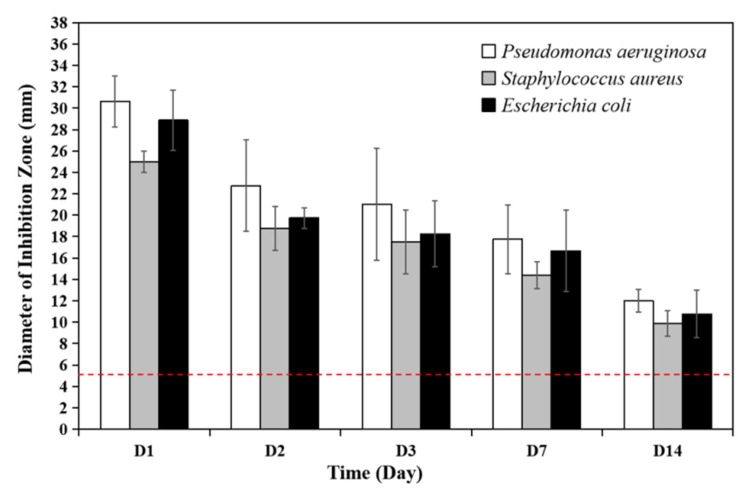
Anti-microbial activities of gentamicin loaded PMMA bone cements were presented by bacterial inhibition zone assay for *Pseudomonas aeruginosa*, *Staphylococcus aureus* and *Escherichia coli*. The dashed line indicates 5 mm, the diameter of the bone cement disc.

The radical polymerization is an exothermic chemical reaction producing heat during the procedure. To evaluate whether the anti-bacterial effects of PMMA bone cements loaded with antibiotics were eliminated by heat produced during polymerization, bacterial inhibition by released antibiotics was examined in vitro. The anti-microbial activity of the basic PMMA bone cement against *Pseudomonas aeruginosa*, *Staphylococcus aureus* and *Escherichia coli* was tested at day 1, 2, 3, 7 and 14, respectively. The results showed that the basic bone cement loaded with gentamicin exhibited great effectiveness in inhibiting all the three bacteria with the diameters of inhibition zones significantly exceeding the control group (PMMA bone cement without antibiotics, 5 mm) (Figure 3, Table 3). Thus, it is obvious that the heat produced during the polymerization of the bone cements did not affect the antibacterial activity of the loaded gentamicin.

### 3.2. Effects of Liquid/Powder Ratio on Antibiotic Elution

After confirming the formulation of basic bone cement loaded with gentamicin, we further intended to improve the antibiotic elution rate from bone cements by varying the basic acrylic bone cement components, such as liquid/powder ratios, radiopacifier (BaSO_4_) ratios, initiator (BPO) ratios and doses of gentamicin.

**Figure 4:**
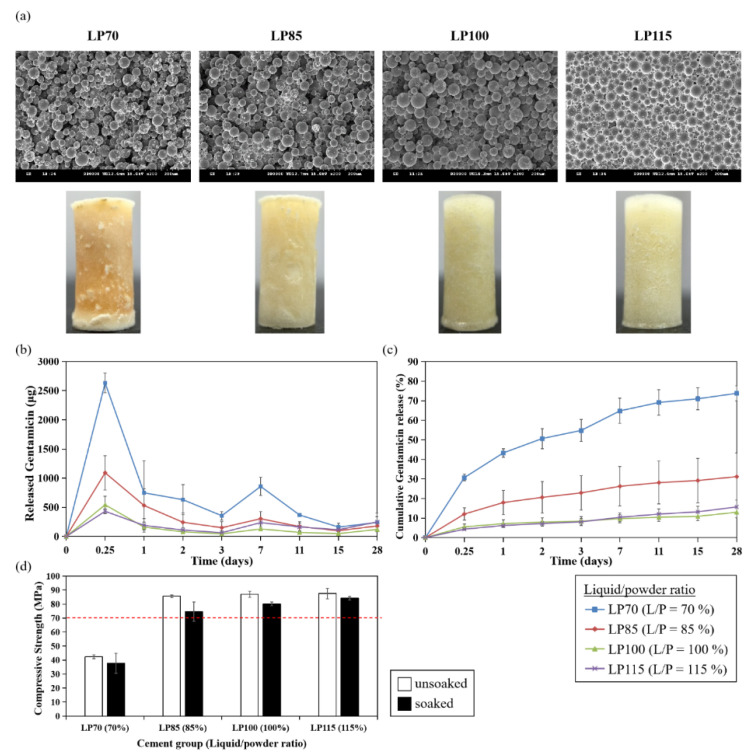
Effects of liquid/powder ratio on antibiotics elution and compressive strength of PMMA bone cement. (**a**) SEM images and morphology of PMMA cement. (**b**) Gentamicin elution profile. (**c**) Cumulative gentamicin release percentage. (**d**) Compressive strength. White bar: unsoaked cement; black bar: PBS-soaked cement. The dashed line indicates the ISO standard for compressive strength (70 MPa).

Firstly, the effect of liquid/powder ratio (monomer-to-polymer ratio) on antibiotic elution was studied. As the original liquid/powder ratio, shown in Table 1, was set as 100% (2 mL/4 g; LP100 in Table 2), ratios of 70% to 115% (LP70, LP85 and LP115 in Table 2) were evaluated for comparison. Table 4 and Figure 4a show the porosity, particle morphology and appearance of ALBC with different liquid/powder ratios. Notably, the porosity of LP70 was 72.3% which was much higher than other groups (12~19.6%; Table 4). Low liquid/powder ratio may result in incomplete polymerization, thus the porosity and pore diameter increased with the decreased liquid/powder ratio in the bone cement. The appearance of bone cement from LP70 was also rough and yellow, while turning smooth and white with the increased liquid/powder ratios.

The kinetics of antibiotic release from bone cements were determined in vitro. Figure 4b,c presents the profiles of gentamicin elution and the cumulative release percentage of gentamicin from PMMA bone cement from day 0 to day 28. All cements showed burst release of the antibiotic during the first day of elution, and the elution rate decreased to sustain a constant drug release over time. This obtained trend was attributed to the rapid dissolution of the antibiotic from the cement surface in the early phase and then the drug was eluted constantly through cracks, gaps, or elution paths from the PMMA matrix [18,19].

The cumulative release percentage of LP100 group (13% at day 28, Figure 4c) represented the drug release behavior of basic bone cement formulation, which was comparable with other studies [13,15]. Among these groups of different liquid/powder ratios, LP70 demonstrated the best cumulative elution of about 73.8% at day 28 and the gentamicin release decreased with the increased liquid/powder ratio (LP85 vs. LP100: 31.2% vs. 13%), though LP100 showed similar gentamicin release behavior with LP115 (LP100 vs. LP115: 13% vs. 15.7%; Figure 4c). The results indicated that altering the monomer-to-powder ratio of ALBC considerably affected the material behavior of the cement, where the lower ratio could considerably enhance the release of gentamicin without adding extra additives, such as xylitol, onto the PMMA cement.

However, the mechanical property of LP70 showed a dramatic fall denoted by the lower compression strength of 42.3 MPa which was below the ISO standards (70 MPa), while LP85, LP100 and LP115 exhibited better compression strength by exceeding 70 MPa of ISO 5883 standards (Figure 4d). The low compressive strength from LP70 may be caused by its large porosity and pore size.

### 3.3. Effects of Radiopacifier Ratio on Antibiotic Elution

**Figure 5:**
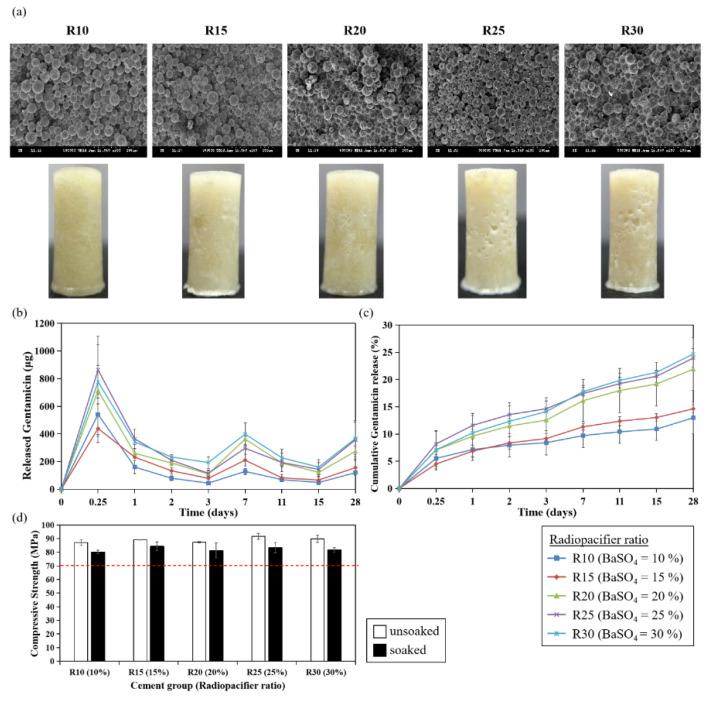
Effects of radiopacifier ratio on antibiotics elution and compressive strength of PMMA bone cement. (**a**) SEM images and morphology of PMMA cement. (**b**) Gentamicin elution profile. (**c**) Cumulative gentamicin release percentage. (**d**) Compressive strength. White bar: unsoaked cement; black bar: PBS-soaked cement. The dashed line indicates the ISO standard for compressive strength (70 MPa).

Following the study of the effects of liquid/ powder ratios, radiopacifiers (BaSO_4_), another component of PMMA bone cement, are needed for monitoring and evenly distributed in the polymer matrix without integrating in the polymer chains. To explore whether radiopacifiers exert effects on antibiotic release, ALBC with different ratios of BaSO_4_ (Table 2; R10 = 10%, R15 = 15%, R20 = 20%, R25 = 25%, R30 = 30%) were tested. With the increased ratio of BaSO_4_ added in the cement, the porosity and pore diameter increased (Table 5). The surface of bone cements with lower ratios of radiopacifier (R10, R15 and R20) was smooth, while a rough surface could be observed with the increased ratios (R25 and R30) (Figure 5a).

Apparently, a higher ratio of radiopacifier enhanced the elution rate and the cumulative release of gentamicin from the bone cement. The percentage of cumulative release of gentamicin was 13%, 14.6%, 21.9%, 24%, 24.7% for R10, R15, R20, R25 and R30, respectively (Figure 5b,c). As shown in Figure 5d, the mechanical property of this sample group was all greater than 70 MPa with no significant difference between different ratios. Thus, the change in the radiopacifier ratio had no effect on the mechanical property of the acrylic bone cement.

### 3.4. Effects of Initiator Ratio on Antibiotic Elution

**Figure 6:**
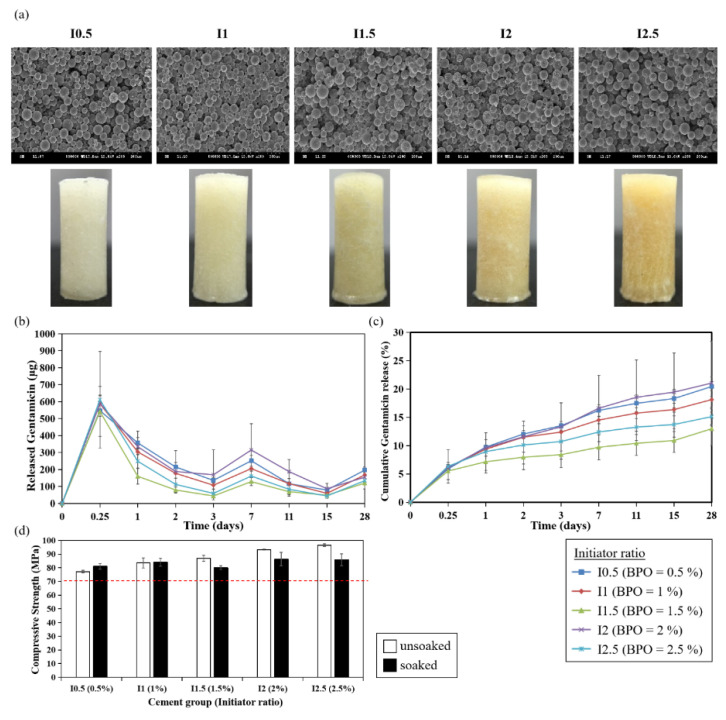
Effects of initiator ratio on antibiotics elution and compressive strength of PMMA bone cement. (**a**) SEM images and morphology of PMMA cement. (**b**) Gentamicin elution profile. (**c**) Cumulative gentamicin release percentage. (**d**) Compressive strength. White bar: unsoaked cement; black bar: PBS-soaked cement. The dashed line indicates the ISO standard for compressive strength (70 MPa).

Initiator BPO triggers the polymerization of MMA monomers by reacting with an activator to form radicals. To explore whether the ratio of the initiator could affect antibiotic release, various percentages of BPO were examined in this section, as listed in Table 2 (I0.5 = 0.5%, I1= 1%, I1.5 = 1.5%, I2 = 2%, I2.5 = 2.5%). Interestingly, bone cement with 1.5% BPO (I1.5) displayed the lowest porosity and pore diameter in this sample group (Table 6). SEM images showed no difference of these particles between cement samples. The color of bone cement became yellow when the BPO ratio was raised (Figure 6a).

The gentamicin elution profile and cumulative release data suggested that the ratio of the initiator exerted no significant effects on gentamicin release, as shown in Figure 6b,c (cumulative release percentage: 20.4%, 18.1%, 13%, 21.1%, 15.1% for I0.5, I1, I2, I2.5, respectively). In contrast, the compression strength was promoted when the ratio of initiator increased (Figure 6d).

### 3.5. Effects of Antibiotic Doses on Antibiotic Elution

**Figure 7:**
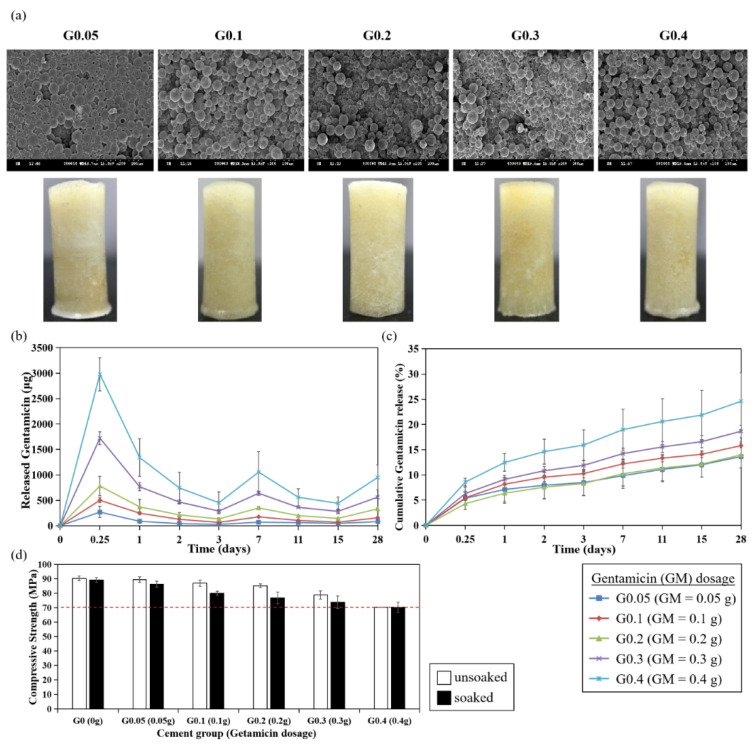
Effects of gentamicin doses on antibiotics elution and compressive strength of PMMA bone cement. (**a**) SEM images and morphology of PMMA cement. (**b**) Gentamicin elution profile. (**c**) Cumulative gentamicin release percentage. (**d**) Compressive strength. White bar: unsoaked cement; black bar: PBS-soaked cement. The dashed line indicates the ISO standard for compressive strength (70 MPa).

Lastly, to study the effects of antibiotic doses on its elution from the bone cement, different concentrations of gentamicin (G0.05 = 0.05 g, G0.1 = 0.1 g, G0.2 = 0.2 g, G0.3 = 0.3 g, G0.4 = 0.4 g) were added and mixed with 4 g of the basic bone cement formulation shown in Table 2. As the added concentration of gentamicin increased, cement porosity and pore diameter increased, while the appearance showed no alteration (Table 7, Figure 7a).

Notably, the cumulative gentamicin release were 13.6% (G0.05), 15.8% (G0.1), 14% (G0.2), 18.7% (G0.3) and 24.6% (G0.4), respectively, suggesting that the elution of gentamicin was positively correlated with its dose (Figure 7b,c). The mechanical property significantly declined with the increased concentration of gentamicin. Although G0.4 group demonstrated great elution of gentamicin, its compressive strength was 69.6/68.3 MPa (PBS unsoaked/soaked), which was below the ISO standards (Figure 7d).

## 4. Discussion

In this present study, different components from basic bone cement compositions, including liquid/powder ratios, radiopacifiers, initiators, and doses of gentamicin, were varied one at a time to explore their effects on the elution of antibiotics from bone cements.

Our results provide strong evidence that porosity of bone cement is positively related to the elution rate of gentamicin. It has been indicated that sustained antibiotic release over a period of time may depend on the penetration depth as determined by the bulk porosity of the cement and antibiotics, which may diffuse through the interconnected series of cracks and voids in the polymer matrix [15,19]. Therefore, it is expected that high porosity would increase the release of antibiotics from ALBC, and this could explain the high elution rate of LP70 group since the porosity was 72.3%, which was much higher than other groups (12~19.6%; Table 4). The porosity of LP100 and LP115 was similar (LP100 vs. LP115: 12% vs. 14.6%; Table 4), resulting in similar gentamicin release behavior (LP100 vs. LP115: 13% vs. 15.7%; Figure 4c). This trend has also been observed in the study of the effect of different radiopacifier ratios on the gentamicin elution rates from the bone cements (i.e., high porosity resulted in high elution rate). It has been mentioned that radiopacifiers could interrupt the polymerizing matrix during the procedure, resulting in the formation of pores to initiate fraction [20,21,22]. Indeed, the porosity and pore size of R15 to R30 listed in Table 5 were higher than R10 (containing 10% BaSO_4_ as basic formulation), which may enhance the elution of antibiotics from cement. In addition to porosity, different mechanisms such as polymeric packing relaxation, polymer erosion, and molecular diffusion can also govern the drug release from polymer matrices. Therefore, different kinetic mathematical models (e.g., Higuchi model, Hixson Crowell model and Korsmeyer-Peppas model) should be fitted to gentamicin cumulative release data to further explore the underlying mechanisms [23].

Compressive strength is one of the important mechanical properties of bone cement. Though LP70 group showed excellent elution rate of gentamicin, the compressive strength from LP70 was low (Figure 4, Table 4) and it may not provide adequate mechanical strength for prosthesis fixation in total joint replacement. However, LP70 still exerts potential for application to other parts of the body where high compressive strength is not required and can be used as a temporary anti-microbial treatment [7]. Pascual et al. suggested that compressive parameters are poorly affected by the liquid/powder ratio [24]. Other studies also showed there was no significant difference in compressive strength when the recommended powder weight (in gram) to liquid volume (in mL) ratio of 2:l was increased to 3:l [25,26]. In this study, the slightly increased compressive strength was observed with PBS-soaked cement containing a higher liquid/powder ratio, which is contrary to the findings from Belkoff et al., who reported that compressive material properties decreased as the ratio of monomer to powder was increased [27]. Whether mechanical properties of bone cement are affected by the liquid/powder ratio remains controversial. Interestingly, several evidences demonstrated that mechanical properties were strongly governed by initiator and activator concentrations [21]. In this present study, we also found that increased initiator/BPO ratios resulted in increased compressive strength (Figure 6d). As the concentrations of BPO and DMPT increased, faster radical formation activates more polymer chain growth simultaneously, accelerating overall polymerization, resulting in increased mechanical strength of the cement cores. Apart from the liquid/powder ratio and initiator, our results indicated that doses of antibiotic could also affect the compressive strength of bone cements (Figure 7d). Although various studies showed that the addition of antibiotics within a certain range of doses did not decrease the compressive strength of bone cements [28,29], Dunne et al. have shown that adding 2, 3, or 4 g of gentamicin to 40 g of Palacos^®^ R cement resulted in a decrease in the compressive strength below 70 MPa [30]. Loading large doses of gentamicin may form clusters, which could act as stress concentrations within the bone cement, resulting in reduced compressive, bending, and fatigue properties of the bone cement.

Instead of powder antibiotics, it has been shown that ALBC loaded with antibiotic liquid solution could significantly improve the efficiency of antibiotic elution. However, the ultimate compressive strength was significantly reduced below the standard in specimens containing liquid antibiotics, suggesting liquid antibiotic-loaded ABLCs may not provide sufficient mechanical strength for use in prosthesis fixation in total joint replacement [7]. Since liquid gentamicin may dilute the liquid monomer and cannot be realistically used to produce high-concentration gentamicin PMMA, gentamicin-impregnated PMMA made with lyophilized liquid gentamicin was examined. Interestingly, the elution rate of lyophilized liquid gentamicin was approximately two times higher than gentamicin powder in preliminary in vitro studies. Mechanical properties of lyophilized liquid gentamicin-loaded PMMA cement still need to be further determined [31].

## 5. Conclusions

**Figure 8:**
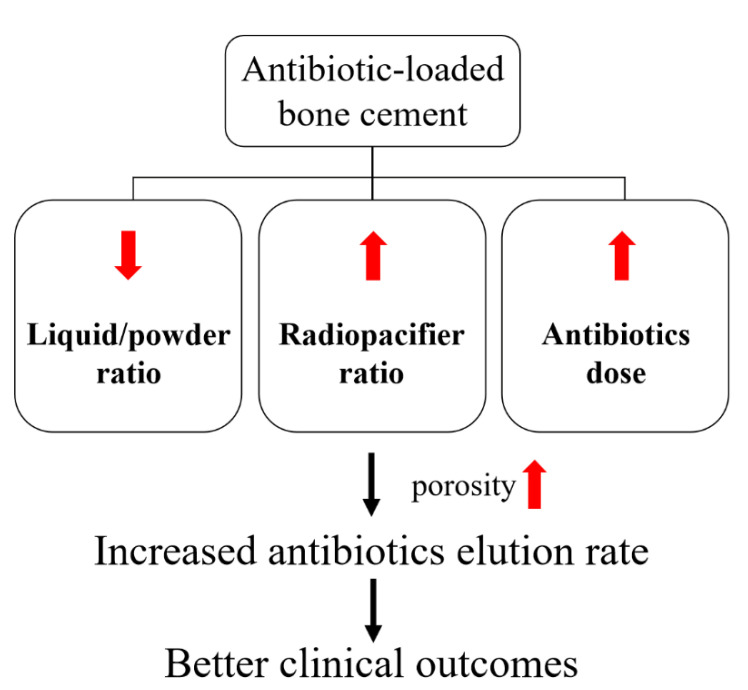
Decreased liquid/powder ratios, increased radiopacifier ratios and higher antibiotic doses may improve the elution rate of antibiotics from ALBC and therefore enhance the clinical efficacy against infection.

In this study, a basic PMMA bone cement was formulated with good biocompatibility and mechanical strength. To enhance the anti-microbial effects of ALBC, we focused on adjusting the range of components in the basic acrylic bone cement. Intriguingly, our results demonstrated that antibiotic elution efficacy rate was attributed to several components of bone cement. Decreased liquid/powder ratio (85%), increased radiopacifier ratio (20~30%) and higher antibiotic concentration (0.3 g gentamicin in 4 g bone cement) led to higher porosity, resulting in better elution of antibiotics without compromising the mechanical strength of the cured bone cement (Figure 8). The results obtained from our study offered precise components that are needed to be considered for developing desirable ALBC, which could be a useful reference for further commercializing the product. In addition, we hope that our findings will also provide insights into controlling antibiotic release from ALBC without the incorporation of extra additives for achieving an effective infection control, which might help in promoting the success rate of the arthroplasty in real-world scenarios.

## Figures and Tables

**Table 1:** Composition of basic PMMA bone cement in this study.

Basic Bone Cement Composition (Liquid/Powder Ratio = 100%)				Antibiotic (0.1 g)
Powder Components (4 g)		Liquid Components (2 mL)		
PMMA (*w*/*w* %)	88.5%	MMA	98.5%	Gentamicin
BaSO4 (*w*/*w* %)	10.0%	DMPT	1.5%	
BPO (*w*/*w* %)	1.5%			

**Table 2:** Composition of tested PMMA bone cements with different parameters.

Parameter	Liquid/Powder %				Radiopacifier					Initiator					Antibiotic				
Sample Groups	LP	LP	LP	LP	R	R	R	R	R	I	I	I	I	I	G	G	G	G	G
	70	85	100	115	10	15	20	25	30	0.5	1	1.5	2	2.5	0.05	0.1	0.2	0.3	0.4
L/P ratio (%)	70	85	100	115	100					100					100				
Liquid (mL)	1.4	1.7	2	2.3	2					2					2				
Powder (g)	4				4					4					4				
PMMA (%)	88.5				88.5	83.5	78.5	73.5	68.5	89.5	89	88.5	88	87.5	88.5				
BaSO_4_ (%)	10				10	15	20	25	30	10					10				
BPO (%)	1.5				1.5					0.5	1	1.5	2	2.5	1.5				
Gentamicin (g)	0.1				0.1					0.1					0.05	0.1	0.2	0.3	0.4

**Table 3:** Measurements (in mm) of the zones of inhibition with bone cements containing gentamicin.

Bacteria ^1^ ZOI (mm)	*Pseudomonas aeruginosa*	*Staphylococcus aureus*	*Escherichia coli*
Day 1	30.63 ± 2.36 mm	25.00 ± 1.00 mm	28.88 ± 2.84 mm
Day 2	22.75 ± 4.27 mm	18.75 ± 2.06 mm	19.75 ± 0.96 mm
Day 3	21.00 ± 5.23 mm	17.50 ± 3.00 mm	18.25 ± 3.10 mm
Day 7	17.75 ± 3.20 mm	14.38 ± 1.25 mm	16.67 ± 3.82 mm
Day 14	12.00 ± 1.08 mm	9.88 ± 1.18 mm	10.75 ± 2.22 mm

^1^ ZOI: zone of inhibition.

**Table 4:** Porosity of PMMA bone cement with different liquid/powder ratios.

Sample Group (Liquid/Powder Ratio)	LP70 (70%)	LP85 (85%)	LP100 (100%)	LP115 (115%)
Total% Porosity	72.3	19.6	12	14.6
Median Pore Diameter (µm)	11.2	7	4.2	4.7

**Table 5:** Porosity of PMMA bone cement with different radiopacifier ratios.

Sample Group (Radiopacifier Ratio)	R10 (10%)	R15 (15%)	R20 (20%)	R25 (25%)	R30 (30%)
Total% Porosity	12	18.1	18.5	24.7	23.1
Median Pore Diameter (µm)	4.2	5.3	7.4	8.5	13.9

**Table 6:** Porosity of PMMA bone cement with different initiator ratios.

Sample Group (BPO Ratio)	I0.5 (0.5%)	I1 (1%)	I1.5 (1.5%)	I2 (2%)	I2.5 (2.5%)
Total% Porosity	18.4	19.5	12	15.5	15.1
Median Pore Diameter (µm)	7	6.3	4.2	5.6	5

**Table 7:** Porosity of PMMA bone cement with different gentamicin concentrations.

Sample Group (Doses of Gentamicin)	G0.05 (0.05 g)	G0.1 (0.1 g)	G0.2 (0.2 g)	G0.3 (0.3 g)	G0.4 (0.4 g)
Total% Porosity	11.6	12	27.9	26	39.3
Median Pore Diameter (µm)	4	4.2	4.9	12.7	14.9

## Data Availability

The data presented in this study are available on request from the corresponding author.
